# Preoperative diagnosis of a recurrent anaplastic lymphoma kinase-negative inflammatory myofibroblastic tumor of the sigmoid colon using EUS-guided fine-needle aspiration: A case report

**DOI:** 10.1097/eus.0000000000000167

**Published:** 2026-07-04

**Authors:** Ning Xie, Yutong Cheng, Tao Wu, Li Miao, Zhe Kang, Haitao Shi

**Affiliations:** 1 Department of Gastroenterology, the Second Affiliated Hospital of Xi’an Jiaotong University, Xi’an, Shaanxi Province, China; 2 Department of Gastrointestinal Surgery, the Second Affiliated Hospital of Xi’an Jiaotong University, Xi’an, Shaanxi Province, China; 3 Department of Pathology, the Second Affiliated Hospital of Xi’an Jiaotong University, Xi’an, Shaanxi Province, China; 4 Department of Nephrology, the Second Affiliated Hospital of Xi’an Jiaotong University, Xi’an, Shaanxi Province, China.

Inflammatory myofibroblastic tumor (IMT) is a rare mesenchymal neoplasm characterized by the proliferation of spindle-shaped myofibroblastic cells within a background of chronic inflammation.^[1]^ While IMTs most commonly arise in the lungs and abdominopelvic soft tissues, colorectal involvement is extremely rare, with the sigmoid colon being one of the least affected sites.^[2]^ Approximately 50%–60% of IMTs show anaplastic lymphoma kinase (*ALK*) gene rearrangements, generally associated with a favorable prognosis.^[3]^ However, ALK-negative tumors, more common in older adults, often demonstrate more aggressive biological behavior and increased metastasis risk.^[4]^ Preoperative diagnosis of IMT is challenging because these tumors typically arise in the submucosa or muscularis propria, making mucosal biopsies nondiagnostic.^[5]^ EUS provides high-resolution imaging and, when combined with fine-needle aspiration (EUS-FNA), allows targeted cytological and immunohistochemical sampling, significantly improving diagnostic accuracy.

A 55-year-old male patient had a history of IMT resection at the splenic flexure 9 years ago. He presented with left lower-quadrant pain and increased stool frequency (6–7 times per day). Computed tomography showed segmental thickening in the distal descending/proximal sigmoid colon with enlarged mesenteric lymph nodes [Figure 1A]. Colonoscopy identified a short stenosis about 20 cm from the anal verge with congested but intact mucosa [Figure 1B]. Standard biopsies showed chronic inflammation without dysplasia or malignancy. EUS demonstrated a 4-cm hypoechoic lesion originating from the submucosa and muscularis propria with preserved mucosa [Figure 1C]. EUS-FNA revealed spindle cells with an inflammatory background, and immunocytochemistry was positive for DES and CD68 and negative for ALK-1, DOG1, and CD117, supporting IMT [Figure 2A]. The patient underwent laparoscopic low anterior resection, and histopathology confirmed ALK-negative IMT with clear margins and uninvolved lymph nodes [Figure 2B, C]. Margins were clear and nodes were uninvolved. Fluorescence *in situ* hybridization detected no *ALK* rearrangement [Figure 3].

**Figure 1.**
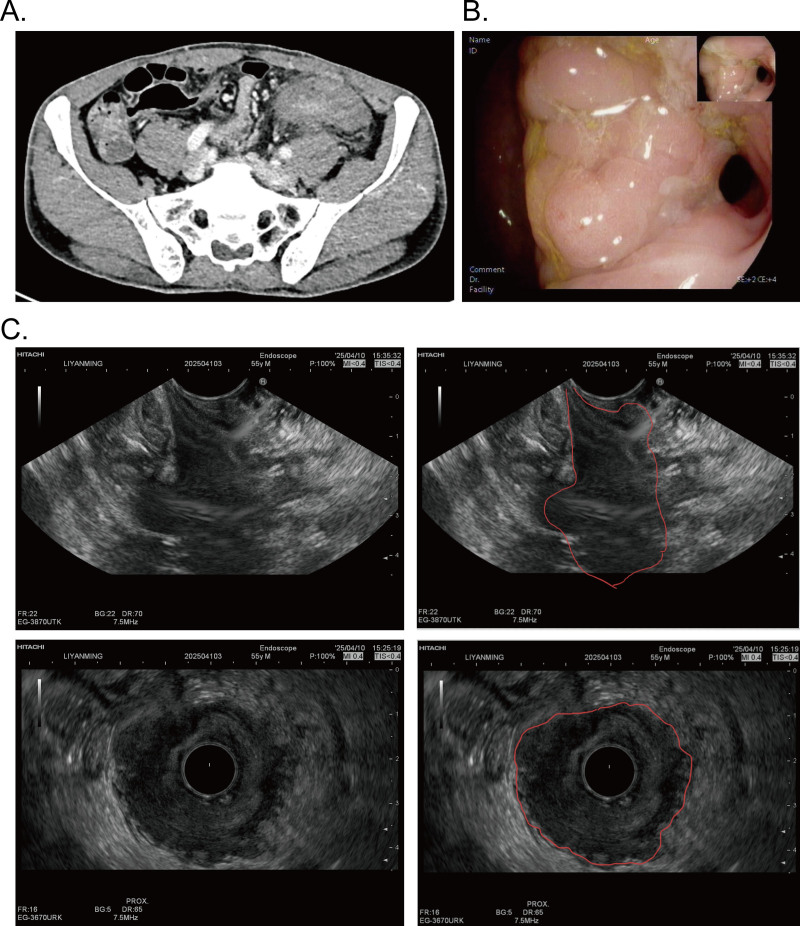
A, In computed tomography imaging, inflammatory myofibroblastic tumors (IMT) presented as well-defined soft tissue masses with heterogeneous enhancement. B, Endoscopically, IMT presented as a submucosal protuberant lesion with an intact overlying mucosa. C, Under EUS, IMT appeared as a heterogeneous hypoechoic mass with relatively well-defined boundaries.

**Figure 2.**
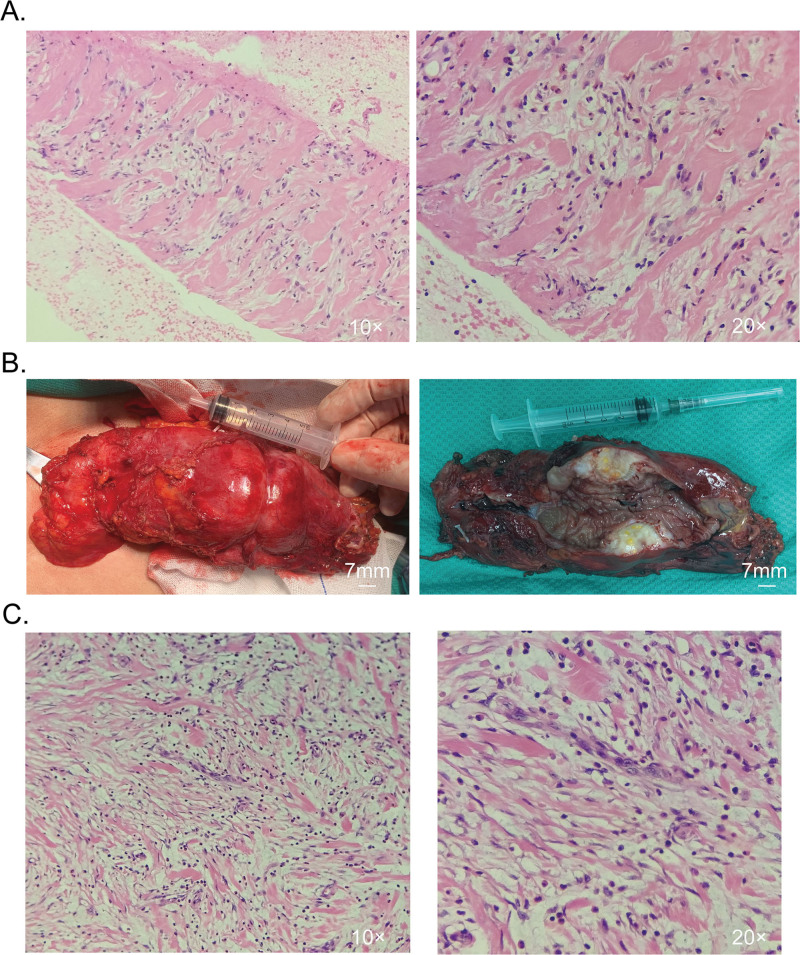
A, Pathological and molecular target testing via EUS-guided fine-needle aspiration (EUS-FNA). B, Tumor resected by laparoscopic radical resection. C, Pathological and molecular target testing of the sigmoid colon resection specimen.

This case demonstrates late recurrence of ALK-negative IMT, highlighting the importance of prolonged surveillance even after a long asymptomatic period postresection. EUS-FNA provided an accurate preoperative diagnosis, facilitating timely curative resection. This case supports the incorporation of EUS-FNA into the diagnostic pathway for colorectal subepithelial tumors, particularly in the context of ALK-negative IMT, and underscores the critical role of EUS-FNA in diagnosing such tumors.

**Figure 3.**
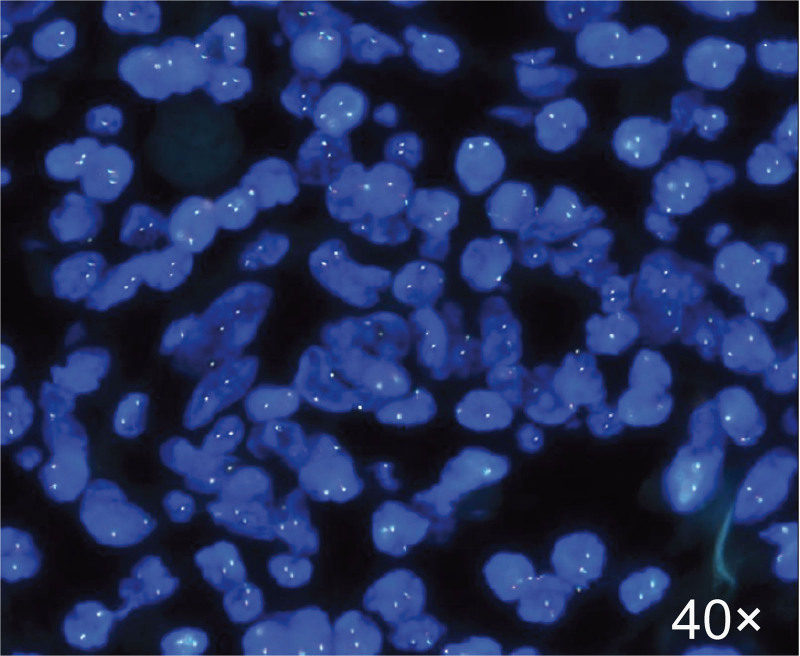
Fluorescence *in situ* hybridization (FISH) analysis showed no *ALK* gene rearrangement. *ALK*, anaplastic lymphoma kinase.

## Ethical Statements

The research protocol was approved by the Ethics Committee of the Second Affiliated Hospital of Xi’an Jiaotong University. Informed consent was obtained from the patient for the publication of his information and imaging.

## Conflicts of Interest

The authors declare that they have no financial conflict of interest with regard to the content of this report.

## Author Contributions

Conceptualization: H. Shi and N. Xie. Writing—original draft preparation and preparing figures: N. Xie and Y. Cheng. Data collection: N. Xie and Y. Cheng. Data curation and preparing tables: T. Wu, L. Miao, and Z. Kang. Writing—reviewing and editing: T. Wu, L. Miao, Z. Kang, and H. Shi. All authors reviewed the manuscript.

## Data Availability Statement

No datasets were generated or analyzed during the current study.
